# Identification and Characterization of MT-1X as a Novel FHL3-Binding Partner

**DOI:** 10.1371/journal.pone.0093723

**Published:** 2014-04-01

**Authors:** Xin Cai, JinFeng Wang, Xin Huang, Wenliang Fu, Wenrong Xia, Minji Zou, YuanYuan Wang, Jiaxi Wang, Donggang Xu

**Affiliations:** Laboratory of Genome Engineering, Beijing Institute of Basic Medical Sciences, Beijing, PR China; Hungarian Academy of Sciences, Hungary

## Abstract

Four and a half LIM domain protein 3 (FHL3) is a member of the FHL protein family that plays roles in the regulation of cell survival, cell adhesion and signal transduction. However, the mechanism of action for FHL3 is not yet clear. The aim of present study was to identify novel binding partner of FHL3 and to explore the underlying mechanism. With the use of yeast two-hybrid screening system, FHL3 was used as the bait to screen human fetal hepatic cDNA library for interacting proteins. Methionine-1X was identified as a novel FHL3 binding partner. The interaction between FHL3 and the full length MT-1X was further confirmed by yeast two-hybrid assay, co-immunoprecipitation and GST pull-down assays. Furthermore,the result demonstrated that MT-1X knockdown promoted the FHL3-induced inhibitory effect on HepG2 cells by regulating FHL3-mediated Smad signaling and involving in the modulation the expression of G2/M phase-related proteins through interaction with FHL3. These findings suggest that functional interactions between FHL3 and MT-1X may provide some clues to the mechanisms of FHL3-regulated cell proliferation.

## Introduction

FHL3 is a member of the four and half LIM domain (FHL) family, which consists of FHL1, FHL2, FHL3, FHL4, and FHL5/ACT. By Northern analysis, FHL1, FHL2, FHL3, and FHL5 were found to be expressed at high levels in a variety of tumor cell lines, including squamous cell carcinoma, melanoma, and leukemia cell lines [1]. The FHL proteins are characterized by four complete LIM domains preceded by an N-terminal half LIM domain [2]. LIM domains are composed of a double zinc finger motif that co-ordinate two zinc ions and are primarily thought to mediate protein-protein interactions [3]. Through interaction with cellular proteins, FHL proteins regulate cellular processes, including proliferation, differentiation, apoptosis, adhesion, migration, transcription, and signal transduction [2].

FHL3 was initially identified in skeletal muscle and reported to regulate transcription factors including CREB,MZF-1 and MyoD by acting as a transcriptional co-activator or co-repressor [4]-[7]. Recently, FHL3 has been shown to play roles in carcinogenesis. It has been demonstrated that FHL3 physically and functionally interact with Smad2, Smad3, and Smad4, important regulators of cancer development and progression, and inhibit human hepatoma cell growth *in vitro* and *in vivo* [8]. Overexpression of FHL3 also has been reported to reduce the proliferation of glioma cells and increase percentage of apoptotic cells [9]. Besides, FHL3 protein has been identified as a negative regulator of HIF-1 activity, implying a mechanism by which it suppresses tumor growth [10]. In addition, FHL3 is involved in the development and progression of breast cancer by suppressing breast cancer cell growth and inducing cell-cycle arrest [11]. Furthermore, our previous study has demonstrated that FHL3 is required for Ang-mediated HeLa cell proliferation by inhibiting nuclear translocation of Ang, suggesting that it may also play an important role in Ang-regulated cancer cell growth and apoptosis [12].

More and more functions of FHL3 have been found, but the underlying mechanisms in different carcinogenesis are not quite clear. In order to explore the mechanisms of FHL3-regulated functions, we used FHL3 as the bait to screen human fetal hepatic cDNA library for its novel interacting proteins in yeast two-hybrid system. Methionine-1X subtype (MT-1X), a member of the methionine, was identified as a novel FHL3 binding partner. The specific interaction between FHL3 and the full length MT-1X was further confirmed by yeast two-hybrid assay, co-immunoprecipitation and GST pull-down assays. The results show that direct interaction between FHL3 and MT-1X exists *in vivo* and *in vitro*. Moreover, MT-1X knockdown promoted the FHL3-induced inhibitory effect on HepG2 cells by regulating FHL3-mediated Smad signaling.

## Materials and Methods

### Materials

Human fetal hepatic MATCHMAKER cDNA library and YEASTMAKER Yeast Transformation system were purchased from CLONTECH Laboratories. Pierce GST Protein Interaction Pull-Down Kit and Pierce Human *In Vitro* protein expression kit for DNA templates were purchased from Thermo scientific. Mouse anti-MT-1X monoclonal antibody was purchased from Novus Biological, CO., Rabbit anti-FHL3 polyclonal antibody was purchased from ProteinTech Group, Inc.

### Cell lines

African green monkey kidney cells (COS-7) (ATCC # CRL-1651), human embryonic kidney cells (HEK 293T) (ATCC # CRL-11268) and human hepatoma cell lines (HepG2) (ATCC # HB-8065) were maintained in Dulbecco's modified Eagle's medium (Hyclone, USA) supplemented with 10% fetal bovine serum, at 37°C with 5% CO_2_.

### DNA constructs

We previously demonstrated that the fourth LIM domain (LIM4) of FHL3 has the auto-activation ability [13]. Therefore, FHL3 mutant with sequential deletion of LIM4 (deletion of 664–840 bp) was amplified using pBV220/FHL3 as template and a forward primer (5′?CTGGTTCATAGAGCGAGTCATTTGACTGTGC?3′) with an *Nde*I site (underlined) and a reverse primer (5′?GAAGATCTGAATTCAATGCACTTAGGTGCAAAGAGTTCTCC?3') with an *Eco*RI site (underlined), respectively. The PCR product was digested with *Nde*I and *Eco*RI, and then inserted into the *Nde*I and *Eco*RI site of vector pAS2-1 of the yeast two-hybrid system (Matchmaker 2, Clontech), resulting in an in-frame fusion of human FHL3 mutants with sequential deletion of LIM4 cDNA downstream with GAL4 DNA-binding domain. The correct reading frame was confirmed by sequencing analysis. The expression vector was named as pAS2-1/FHL3d4.

### Testing pAS2-1/FHL3d4 plasmid for auto-activation

It is crucial to test each BD-bait plasmid construct for auto-activation of reporter gene promoters before performing the two-hybrid screen. To check for auto-activation, the yeast strain Y190 was transformed with pAS2-1/FHL3d4 using lithium acetate method as described in protocol. Transformed cells were plated on synthetic dropout medium minus tryptophan (SD/-Trp, bait plasmid contains a tryptophan as a selection marker for growth control). Auto-activation of FHL3d4 was screened by β-galactosidase assay using a filter-based assay.

### Library screening

A human fetal hepatic cDNA library in pACT2 plasmid was screened with bait plasmid pAS2-1/FHL3d4 using a large scale sequential transformation method according to the manufacturer's instructions. Yeast cells of Y190 strain harboring pAS2-1/FHL3d4 were transformed with 50 μg of library cDNA and plated on medium lacking tryptophan, leucine and histidine (SD/-Leu -Trp -His) containing 25 mM 3-amino-1,2,4-triazole(3-AT) and incubated for 4–7 days at 30°C. Then, colonies were assayed for β-galactosidase activity. Following a second round of selection on the same medium and removing the valid AD plasmids, the colonies that activated both reporter genes in Y190 strain were extracted and transformed into *Escherichia coli* DH5α cells for further analysis.

### Confirmation of positive clones

After checking AD-cDNA plasmids for auto-activation, AD-cDNA plasmids were transformed into Y190 strain harboring pAS2-1/FHL3d4 plasmid to assay the activation of reporter genes. The positive AD-cDNA clones were further characterized by DNA sequencing and these DNA sequences were analyzed by BLAST search for GenBank database.

### Confirmation of interaction between full length MT-1X and FHL3d4 by yeast two-hybrid assay

The cDNA coding for MT-1X (GenBank accession no. NM005952) was amplified from the cDNA library of the human fetal hepatic using a forward primer (5′?GCGGATCCCCATGGACCCCAACTGCTCCTGC?3′) with an *Bam*HI site (underlined) and a reverse primer (5′?GCGAATTCTCAGGCACAGCAGC TGCACTT?3') with an *Eco*RI site (underlined) and then subcloned into pACT2. pAS2-1/FHL3d4 and pACT2/MT-1X were transformed into Y190 strain. Transformation of the plasmids was carried out according to Table 1.

**Table 1:** The corresponding plasmids and culture medium for confirmation of interaction between full length MT-1X and FHL3d4.

Transformed plasmids for control	Culture medium
pAS2-1/FHL3d4 and pACT2/MT-1X	SD/-Leu-Trp, SD/-Leu-Trp-His
pAS2-1/FHL3d4 and pACT2	SD/-Leu-Trp, SD/-Leu-Trp-His
pAS2-1 and pACT2/MT-1X	SD/-Leu-Trp, SD/-Leu-Trp-His
pAS2-and pACT2	SD/-Leu-Trp, SD/-Leu-Trp-His
pAS2-1/FHL3d4	SD/-Trp
pACT2/MT-1X	SD/-Trp

### Co-immunoprecipitation

The full length cDNA encoding FHL3 was amplified by PCR from pBV220/FHL3 which was previously constructed in our laboratory, and subcloned into pCMV-HA to generate HA-tagged fusion protein expression vector. The cDNA fragment of MT-1X was generated by PCR using pACT2/MT-1X as a template, and inserted into pCMV-Myc to generate Myc-tagged fusion protein expression vector. 293T cells (7.5×10^6^) were co-transfected with pCMV-HA/FHL3 and pCMV-Myc/MT-1X using Lipofectamine 2000 in a 10 cm dish. After 30 h, 293T cells were harvested and lysed in a Triton lysis buffer consisting of 20 mM Tris-HCl, pH7.6, 120 mM NaCl, 2 mM EDTA, 1% Triton X-100, 10% glycerol with 1 mM Na_3_VO_4_, 1 mM DTT, and protease inhibitor cocktail. The cell lysates were incubated at 4°C for 30 min and centrifuged at 12,000 rpm for 10 min. Lysates were immunoprecipitated with anti-Myc antibody (California Bioscience, USA) or control anti-IgG antibody (California Bioscience, USA) for 4 h at 4°C on a rotor, and then protein A/G–Agarose beads (Santa Cruz) were added and further incubated for 16 h. After incubation, the samples were centrifuged for 5 min at 3000 rpm. The protein A/G–Agarose pellets were then washed and boiled in 2×SDS sample buffer. Immunocomplexes were analyzed by SDS–PAGE and Western blot using anti-myc antibody and anti-HA antibody (California Bioscience).

### Glutathione S-transferase (GST) pull-down assays

The full length cDNA encoding FHL3 was amplified by PCR from pBV220/FHL3 and subcloned into pGEX-4T-1 (Amersham Biosciences, USA). The recombinant protein GST-FHL3 and protein GST were individually expressed in *E. coli* DH5α cell. Cleared lysates were prepared and the soluble fusion proteins were purified by GSTrap FF column (Amersham Biosciences, USA) and tested by Western blot using anti-GST monoclonal antibody (Kangwei, China). The cDNA for MT-1X was amplified by PCR from pACT2/MT-1X, inserted into pT7CFE1-CHis (Thermo) to generate His-tagged fusion protein expression vector pT7/MT-1X, and then translated into protein. Purified GST–FHL3 and GST were individually incubated with Glutathione Agarose for approximately 4 h. Then the Glutathione Agarose was collected by centrifugation, washed five times with GST pull-down lysis buffer and TBS (1:1). Then, His-MT-1X was added to Glutathione Agarose and incubated at 4 °C for overnight with gentle rocking. Glutathione Agarose was washed five times and resuspended in SDS–PAGE sample buffer. After boiling, the bound proteins were analyzed by 15% SDS–PAGE followed by Western blotting using anti-His antibody. His-MT-1X protein was used as controls.

### MTT assay

Various concentrations of rhFHL3 (Peprotech, USA) were added to 96-well plates containing 5,000 cells/well in transfected with siMT-1X or siNC cells. After incubation for 48 h, cell growth was measured by MTT (3-(4,5-dimethylthiazolyl-2)-2, -diphenyltetrazoliumbromide) proliferation assay. Absorbance at 570 nm was measured using a Bio-Rad model 550 microplate reader. The data represent the means of three independent experiments. The cDNA target sequences of siRNAs for MT-1X were: 5′- GGCAAUAAAUUCAUCUAGATT - 3′ (sense) and 5′- UCUAGAUG AAUUUAUUGCCTT - 3′ (antisense). Negative control (Nc) siRNA was included (GenePharma, Shanghai, China).

### Transfections and Western blot analysis

Human hepatoma HepG2 cells were routinely cultured in Dulbecco's modified Eagle's medium (DMEM) supplemented with 10% fetal bovine serum at 37 °C in humidified atmosphere of 5% CO_2_ in air. For transfection, cells were seeded in 96-well or 6-well plates in triplicate containing DMEM supplemented with 10% fetal bovine serum. The cells were transfected with the indicated siRNA or plasmids using Lipofectamine 2000 according to the manufacturer's protocol (Invitrogen). 48 h after treatment with siRNA or plasmids at 37°C , cells were washed twice with ice-cold phosphate-buffered saline (PBS) and lysed in RIPA lysis buffer (Applygen, China) for 15 min on ice. Cell lysates were then clarified by microcentrifugation at 12,000×g for 10 min at 4°C. Equal amounts of protein (40 μg) was quantified by Lowry protein assaying kit (Keygen, Beijing, China), and separated by 12% sodium dodecyl sulfatepolyacrylamide gel electrophoresis (SDS-PAGE) and electrophoretically transferred to polyvinylidene fluoride (PVDF) membranes (Millipore, Bedford, USA). Membranes were then blocked with PBST (PBS with 0.05% Tween 20) containing 5% non-fat dry milk for 1 h at room temperature and followed by incubation with monoclonal antibodies against pSmad2 (Ser465/467) and Smad2, Smad4, P21, CyclinB1 and CyclinD1 (Cell Signaling Technology, USA), His and GAPDH (1:1,000) (ZhongShan Ltd., China) and MT-1X antibodies (Novus, USA), polyclonal antibodies against FHL3 (ProtechTech, USA) overnight at 4°C, respectively. Membranes were then washed with PBST for three times and followed by incubation with HRP-conjugated anti-mouse or HRP-conjugated anti-rabbit immunoglobulin G (IgG) (ZhongShan jinqiao, Beijing, China) at room temperature for 1 h. Finally, the membrane was analyzed using the enhanced chemiluminescence detection system (Amersham Pharmacia Biotech, Canada).

## Results

### A novel binding partner of FHL3d4 is discovered by yeast two-hybrid screening assay

**Figure 1:**
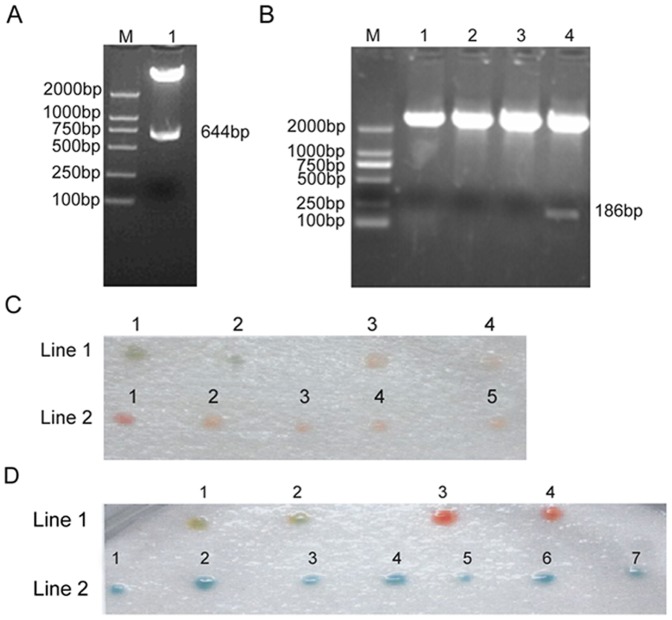
The interaction between full length MT-1X and FHL3 by yeast two hybrid assay. (A) Identification of recombinant plasmid pAS2-1/FHL3d4 by restriction analysis. M:DNA marker; Lane1: Digested pAS2-1/FHL3d4 plasmid with *Nde*I and *Eco*RI. (B) Identification of recombinant plasmid pACT2/MT-1X by restriction analysis. M:DNA marker; Lane1-4: Digested pACT2/MT-1X plasmid with *Bam*HI and *Eco*RI. (C) Detection of MT-1X auto-activation by yeast two hybrid analysis. Line1:1,2: positive control; Line1: 3,4: negative control; Line2:1-5:transform pACT2/MT-1X. (D) Coloration analysis to verify the interaction between full length MT-1X and FHL3. Line1:1,2:positive control; Line1: 3,4: negative control. Line2:1–7: Co-transform pAS2-1/FHL3d4 and pACT2/MT-1X.

The previous results suggested that the fourth LIM domain (LIM4) of FHL3 was the key transactivation domain of FHL3 [13]. Therefore, FHL3 mutant with sequential deletion of LIM4 (deletion of 664–840 bp) was amplified by PCR using pBV220/FHL3 as template, and then inserted into the plasmid pAS2-1 to construct the recombinant plasmid pAS2-1/FHL3d4, which was subsequently confirmed by restriction analysis (Fig. 1A). The FHL3d4 construct did not auto-activate the reporter genes. Then, in yeast two-hybrid screening, we used FHL3d4 as bait protein and obtained 47 positive clones in three deficiency culture medium by β-galactosidase chromatographic analysis. After removing invalid AD plasmids, 34 potential interactors were identified which had no auto-activation and were reintroduced into yeast cells to confirm the interaction with pAS2-1/FHL3d4 bait plasmid. Finally, the colonies were all plasmid-positive. Sequence analysis indicated that only one AD-positive plasmid could find homologous sequence which had 68% identities with Methionine-1X subtype (MT-1X) and encoded amino acids 28–83 MT-1X. Therefore, MT-1X was chosen for further study.

### The interaction between full length MT-1X and FHL3d4 is confirmed by yeast two-hybird assay

To identify the results of yeast two-hybrid screening, a 186 bp sequence of MT-1X cDNA was amplified by PCR from a cDNA library of human fetal hepatic and then inserted in pACT2. The recombinant plasmid pACT2/MT-1X (Fig. 1B, lane4) was identified by restriction analysis and confirmed by sequencing (Fig. 1B). Yeast two hybrid assay confirmed that the recombinant plasmid pACT2/MT-1X had no auto-activation (Fig. 1C). The coloration analysis showed only co-transforming pACT2/MT-1X and pAS2-1/FHL3d4 could activate the reporter gene (Fig. 1D). The results demonstrate that the full length MT-1X interacts with FHL3.

### FHL3 interacts with MT-1X *in vivo*

**Figure 2:**
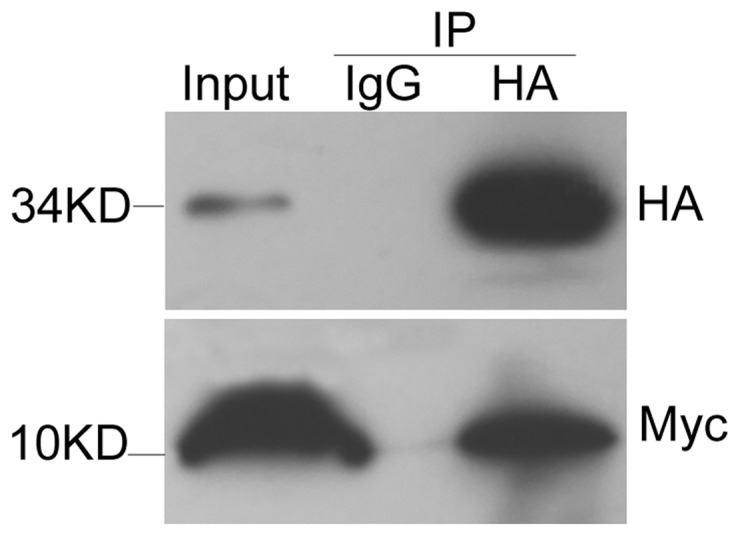
The interaction between FHL3 and MT-1X *in vivo*. 293T cells were transfected by Myc-MT-1X with HA-FHL3. Lane 1: lysate of cells transfected with Myc-MT-1X and HA-FHL3. 2: IP of cells with anti-IgG monoclonal antibody as negative control. 3: IP of cells with anti-Myc monoclonal antibody; After 30 h, total cell lysates were analyzed before co-immunoprecipitation to verify expression of Myc-MT-1X and HA-FHL3 (lane 1). After co-immunoprecipitation with anti-Myc tag antibody, MT-1X was detected only in the presence of immune complexes of Myc-FHL3 (lane 3).

In order to confirm the results of yeast two-hybrid screening and the interaction of FHL3 with MT-1X *in vivo*, co-immuoprecipitation assays were performed in HEK 293T cells. HA-FHL3 and Myc-MT-1X were co-expressed transiently, then expression of recombinant FHL3 and MT-1X was confirmed by Western blotting using anti-HA and anti-Myc antibody respectively. As a result, anti-HA antibody was able to precipitate Myc-MT-1X protein only in the presence of HA-FHL3. As a control, the anti-IgG antibody did not precipitate Myc-MT-1X. It suggests that MT-1X could physically interact with FHL3 *in vivo* (Fig. 2).

### The direct interaction between FHL3 and MT-1X *in vitro* is verified by GST pull-down assay

**Figure 3:**
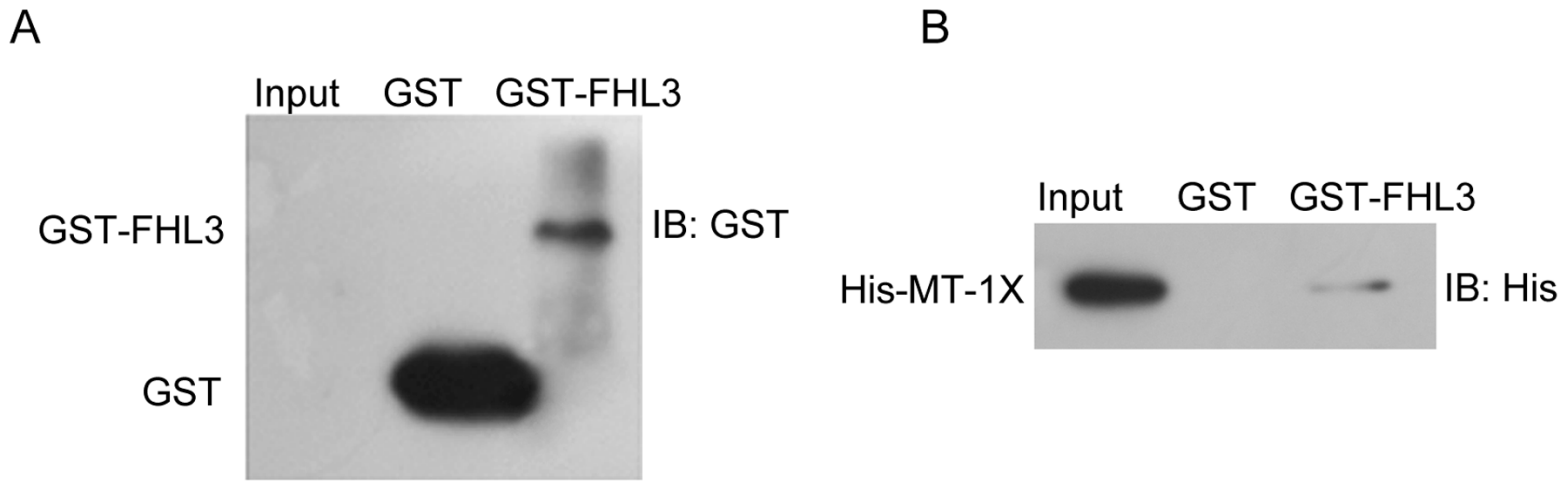
GST pull-down assay demonstrating *in vitro* binding between MT-1X and FHL3. (A) Purification of GST-FHL3 protein. Purification of GST protein (Lane1) and GST-FHL3 protein (Lane2) by Western blot analysis using anti-GST monoclonal antibody. (B) GST pull-down assay. Lane1 demonstrated the signal from purified His-MT-1X. GST protein and GST-FHL3 fusion protein were mixed with purified His-MT-1X (Lane2 and 3) and precipitated with Glutathione Agarose followed by Western blot analysis.

Purified GST-FHL3 and GST protein were confirmed by Western blotting (Fig. 3A). His-MT-1X protein was prepared from *in vitro* transcription/translation reaction, and then individually mixed with Glutathione Agarose bound to either GST protein or the GST–FHL3 fusion proteins. The GST–FHL3 protein was bound to the His-MT-1X. At the same time, expression of His-MT-1X was confirmed by Western blotting using anti-His antibody as a positive control and the binding of His-MT-1X to GST alone was not observed (Fig. 3B). Thus the results demonstrate the direct interaction between FHL3 and MT-1X observed in the yeast two-hybrid screen.

### Knockdown of MT-1X promotes the FHL3-dependent inhibitory effect on HepG2 cells

The effect of FHL3 on human HepG2 hepatoma cells was examined by MTT assay. FHL3 could inhibit the proliferation of HepG2 cells at 10 ng/ml (*p*<0.05). The inhibitory effect of FHL3 on the proliferation of HepG2 cells displayed a dose-dependent manner from 10 to 1250 ng/ml (*p*<0.01), and was saturated above 250 ng/ml. The result was similar to that previously reported [8], [13].

**Figure 4:**
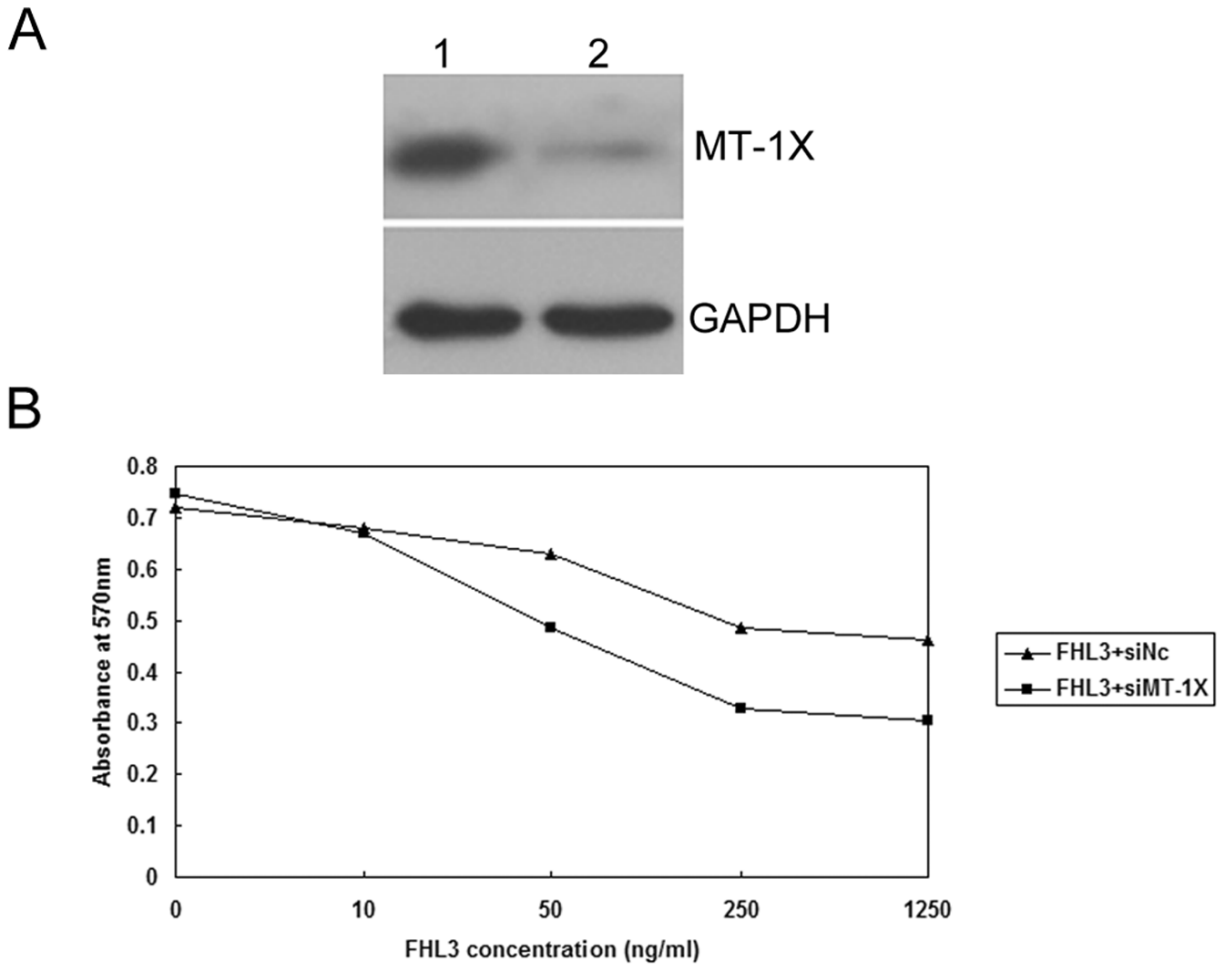
Knockdown of MT-1X promoted the FHL3-dependent inhibitory effect on HepG2 cell. (A) The expression of MT-1X protein of HepG2 cells transfected by small interference RNA against MT-1X or negative control siRNA determined by western blot. 1, Negative control siRNA; 2, siMT-1X. GAPDH was used as loading control. The transfection efficiency of HepG2 is ∼30%. (B) Indicated amounts of rhFHL3 were added to 96-well plates containing 5,000 cells/well in transfected with siMT-1X or siNC cells. After incubation for 48 h, cell growth was measured by MTT assay. The data represent the means of three independent experiments.

Given that FHL3 interacts with MT-1X, we investigate whether MT-1X affects FHL3-dependent inhibition of HepG2 cell growth. As MT-1X was highly expressed in HepG2 cells, using small interfering RNA strategy, we found that knockdown of MT-1X effectively decreased its expression as compared with negative control (Nc siRNA) (Fig. 4A). After confirmation of efficient knockdown of MT-1X, transfected cells were stimulated with FHL3 for 48 h in order to investigate the role of MT-1X in FHL3-dependent inhibition of HepG2 cells growth. The result of MTT analysis indicated that knockdown of MT-1X promoted the FHL3-dependent inhibitory effect on HepG2 cells (Fig.4B). These data suggest that MT-1X may be involved in the regulation of FHL3-mediated signaling pathway through interaction with FHL3.

### MT-1X involves in the FHL3-mediated Smad signaling

**Figure 5:**
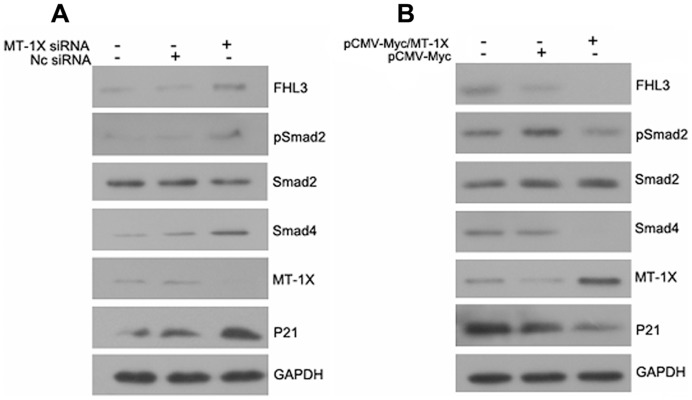
MT-1X involved in the FHL3-mediated TGF-β-like response. (A) HepG2 cells were transiently transfected with NC siRNA or MT-1X siRNA as indicated, and cell lysates were analyzed by Western blot analysis with the indicated antibodies. (B) HepG2 cells were transiently transfected with pCMV-Myc/MT-1X or pCMV-Myc expression vector as indicated, and cell lysates were analyzed by Western blot analysis with the indicated antibodies.

To determine whether the MT-1X influences the growth of HepG2 cells by regulating FHL3-mediated signaling pathways, we primarily focused on the effect of MT-1X on the FHL3-mediated Smad signaling in HepG2 cells. The result of western blotting analysis indicated that knockdown of endogenous MT-1X expression could up-regulate the expression level of FHL3. Moreover, knockdown of MT-1X enhanced phosphorylation of the cytoplasmic Smad2 protein (Ser465/467) and the expression of Smad4 protein, while the protein level of total Smad2 was unchanged. Furthermore, we observed that knockdown of MT-1X induced a up-regulation of tumor suppressor gene p21 in HepG2 cells (Fig. 5A). In contrast, overexpression of MT-1X in HepG2 cells decreased phosphorylation of the cytoplasmic Smad2 protein (Ser465/467) and the expression of Smad4 protein, as well as reduced the expression of p21 (Fig. 5B). These data suggest that MT-1X may be involved in the regulation of Smad signaling through interaction with FHL3.

### MT-1X modulates the expression of G2/M phase-related protein in hepatoma Cells

**Figure 6:**
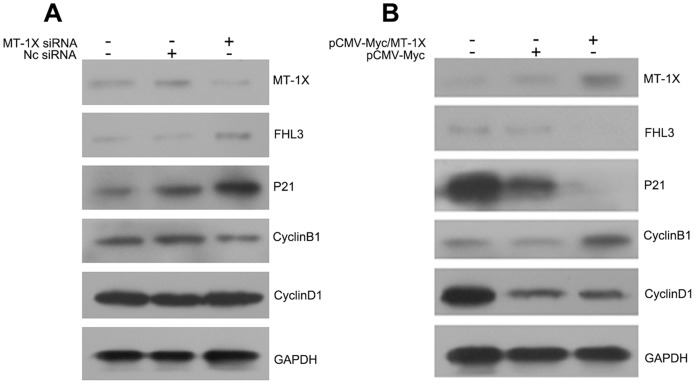
MT-1X regulated expression of cell cycle-related proteins. (A) Extracts from HepG2 cells transfected with MT-1X siRNA were used for Western blot analysis with the indicated antibodies. (B) Extracts from HepG2 cells transfected with MT-1X expression vector were used for Western blot analysis with the indicated antibodies.

To elucidate the molecular mechanism by which MT-1X expression regulated hepatoma cells growth, we investigated the effect of MT-1X on the expression of several cell cycle-related proteins by immunoblotting. Knockdown of endogenous MT-1X in HepG2 cells enhanced the expression of p21 that functions as a negative regulator of cell cycle progression at G1 and G2 [14], and repressed the expression of the G2/M-phase marker cyclin B1 [15] (Fig. 6A). In contrast, overexpression of MT-1X in HepG2 cells reduced the expression of p21 and increased the expression of cyclin B1 (Fig. 6B). However, both knockdown of endogenous MT-1X and overexpression of MT-1X had no significant effect on the protein level of cyclinD1 ,compared with control HepG2 cells (Fig. 6). These data suggest that MT-1X may be involved in the modulation the expression of G2/M phase-related proteins through interaction with FHL3 in HepG2 Cells.

## Discussion

FHL3 is a member of the family of LIM proteins and is involved in myogenesis, cytoskeleton reconstruction, cell growth and differentiation. Recently, FHL3 has been shown to play roles in carcinogenesis [8]–[12]. However, the detailed mechanisms of its actions are still not well-defined.

We started from the interaction proteins of FHL3 to explore the possible mechanism of FHL3 in cell proliferation. Until now, there have been a few proteins known to interact with FHL3, such as Actin, CDC25B2, MZF-1, MyoD, and Sox15 [6], [15]–[19]. In yeast two-hybrid screening, we used FHL3 as bait protein and identified Methionine-1X as a novel binding partner. The interaction between FHL3 and the full length MT-1X was confirmed by yeast two-hybrid assay. The specific and direct interaction *in vivo* and *in vitro* was verified by co-immunoprecipitation and GST pull-down. Moreover, MT-1X is involved in the FHL3-induced inhibitory effect on HepG2 cells by regulating FHL3-mediated Smad signaling, accompanied by increasing of the cell cycle regulator cyclinB1 and inhibition of the cyclin-dependent kinase inhibitor p21.

Metallothioneins (MTs) belong to the group of intracellular cysteine-rich, metal-binding proteins that have been found in bacteria, plants, invertebrates and vertebrates [20]–[22]. In mammals, MTs contain 61–68 amino acids, and among them 20 are cysteines [23], [24], which are characterized by low molecular weight (6–7 kDa). In humans, the MT genes are located on chromosome 16 in a cluster and involve 16 identified genes, from which five are pseudogenes [25]. Although the MT-II, MT-III and MT-IV proteins are encoded by a single gene, the MT-I protein comprises many subtypes encoded by a set of 13 MT-I genes. The known active MT-I genes are MT-IA,-IB, -IE, -1F, -IG, -IH,-IM and -IX. The rest of the MT-I genes (MT-1C,-1D,-1I,-1J and 1L) are pseudogenes that are not expressed in humans [25].

A lot of studies have shown that MT proteins are involved in many pathophysiological processes, including metal ion homeostasis and detoxification, protection against oxidative damage, cell proliferation and apoptosis, drug and radiotherapy resistance and several aspects of the carcinogenic process [26], [27]. Although MT showed increased expression in various human tumors of the breast, kidney, lung, nasopharynx, ovary, salivary gland, testes, thyroid and urinary bladder [26], [27], which was downregulated in certain tumors such as hepatocellular carcinoma (HCC), prostate and colorectal cancer [28]. Further studies have shown that down-regulation of MT synthesis in hepatic tumors may be related to hypermethylation of the MT-promoter or mutation of other genes such as the p53 tumor suppressor gene [29], [30]. However, the possible significance of the downregulation of MT-1X and MT-2A in HCC is presently unknown due to the scarcity of studies on human cells and tissues [31]–[33].

Recently, it has been shown that FHL1, FHL2, and FHL3 physically and functionally interact with Smad2, Smad3, and Smad4, important regulators of cancer development and progression, and inhibit human hepatoma cell growth *in vitro* and *in vivo* [8]. Given that the role of FHL3 in human hepatoma cell, we sought to determine whether MT-1X could play a role in the regulation of FHL3-mediated human hepatoma cell growth. In the current study, we showed that knockdown of MT-1X promoted the FHL3-dependent inhibitory effect on HepG2 cells. Moreover, using small interfering RNA strategy and overexpression of MT-1X, we found that MT-1X was involved in the negative regulation of Smad signaling by controlling the expression level of FHL3 and regulating the expression of p21 in HepG2 cells.

The cyclin-dependent kinase inhibitor p21, the first identified inhibitor of cyclin/CDK complexes, has been shown to be required for the integrity of G1 and G2 checkpoints [34], [35]. Therefore, the cell cycle proteins were analyzed to elucidate the mechanisms by which MT-1X promoted the FHL3-dependent inhibitory effect on HepG2 cells. Cyclin B1 plays an important role in progression of various cancers. The cyclinB1/Cdk1 complex is the primary regulator of the transition from G2 to M phase [36]. Without synthesis of cyclinB1 before the G2/M transition, Cdk1 remains inactive, and the cell cannot enter mitosis, resulting in cell cycle arrest at the G2 phase [37]. Our data suggests that MT-1X inhibition in HepG2 cells can cause G2/M phase arrest by decreasing its protein level. Consistent results were obtained in HepG2 cells transfected with MT-1X expression vector. Cyclin D1/CDK4/6 regulates G1/S transition. However, protein levels of cyclinD1 presented no significant difference between HepG2 cells transfected with MT-1X expression vector or siMT-1X and control HepG2 cells in our study. Together with these observations, our findings suggest that MT-1X regulates cell cycle arrest at the G2 phase by regulating the function of FHL3 in HepG2 cell as a FHL3 interactor. However, the exact mechanism of how MT-1X affects FHL3 molecules involved in proliferation is not clear and should be further investigated.

There have been several reports on the overexpression and specific staining pattern of MT in human breast cancer. Further studies suggest that MT overexpression is associated with significantly poorer prognosis and increased recurrence in invasive ductal breast carcinomas [30]–[32]. However, the exact functions mechanism of MT-1X in breast cancer remains unknown. By establishing a link between MT-1X and FHL3, our findings suggest that FHL3 may be involved in the regulating the function of MT-1X in breast cancer cell as a MT-1X interactor.

In summary, by establishing a link between MT-1X and FHL3, this study highlights MT-1X as a potential candidate for investigating the molecular mechanisms of cancer cell anti-apoptotic activity and also novel useful molecular targets for cancer therapy.
